# Molecular Identification of Parasitic Protozoa *Sarcocystis* in Water Samples

**DOI:** 10.3390/vetsci9080412

**Published:** 2022-08-05

**Authors:** Živilė Strazdaitė-Žielienė, Agnė Baranauskaitė, Dalius Butkauskas, Elena Servienė, Petras Prakas

**Affiliations:** Nature Research Centre, Akademijos Str. 2, LT-08412 Vilnius, Lithuania

**Keywords:** *Sarcocystis*, farm animals, *cox1*, molecular identification, methodology optimization, water samples

## Abstract

**Simple Summary:**

Members of the genus *Sarcocystis* are protozoan parasites having two-host prey–predator cycle. These parasites are widespread in farm animals. *Sarcocystis* species are characterized morphologically in intermediate hosts, and these parasites are identified in definitive hosts by molecular methods. Thus far, only few studies have been conducted on *Sarcocystis* parasites in environmental samples. The aim of the present work was to evaluate several sample preparation and polymerase chain reaction methods for the identification of several *Sarcocystis* species in water samples. Overall, 114 samples collected from various water sources, ponds, canals, lakes, lagoons, and rivers in Lithuania were tested for the presence of *Sarcocystis* spp. Based on molecular methods, eight *Sarcocystis* species, *S. bovifelis*, *S. cruzi*, *S. hirsuta*, *S. arieticanis*, *S. tenella*, *S. capracanis*, *S. bertrami*, and *S. miescheriana*, were identified. The main intermediate hosts of detected *Sarcocystis* parasites are cattle, sheep, goats, horses, and pigs. Further, more sensitive molecular techniques are needed for the development of the diagnosis of *Sarcocystis* species in water bodies.

**Abstract:**

*Sarcocystis* parasites are among the most common parasitic protozoa in farm animals. So far, the diversity of these parasites has been mainly studied in animal carcasses by morphological or molecular methods. Research on parasitic protozoa in environmental samples is scarce due to the lack of an appropriate methodology and low concentrations of parasites. For these reasons, there is a paucity of validated methods for *Sarcocystis* identification from environmental samples. Therefore, the present study aims to investigate various molecular methods for *Sarcocystis* parasite identification in water samples. In the present study, the sample volume, sporocysts isolation, and various conventional PCR were evaluated, and species-specific primers for the identification of different *Sarcocystis* species have been developed. Of the methods studied, based on data the most appropriate method for the identification of analyzed *Sarcocystis* spp. in water bodies is nested PCR, using species-specific primers targeting the *cox1* gene. *Sarcocystis* DNA was detected in 111 out of 114 (97.4%) samples. This paper represents the first identification of *S*. *bovifelis*, *S*. *cruzi*, *S*. *hirsuta*, *S*. *arieticanis*, *S*. *tenella*, *S*. *capracanis*, *S*. *bertrami*, and *S*. *miescheriana* by PCR and sequencing in environmental water samples. Our pilot study is useful in developing techniques for the identification of *Sarcocystis* species from water samples.

## 1. Introduction

*Sarcocystis* parasites are members of the Apicomplexa phylum that infect mammals, birds, and reptiles. They have an obligatory life cycle of two hosts, intermediate and definitive, that implies a predator–prey interaction. Sarcocysts of these parasites are mainly formed in muscle tissues of intermediate hosts (asexual stage), while sporocysts develop in the intestine of the definitive host and are spread into the environment in feces (sexual stage) [1]. Meat of farm animals showing an eosinophilic myositis related to the presence of *Sarcocystis* spp. is withdrawn from the market [2]. Furthermore, intense *Sarcocystis* infection might result in reduced meat, wool, and milk production, causing huge losses in the livestock industry annually [2,3].

To date, the muscle tissues of intermediate hosts have been studied mainly for the purpose of identifying different *Sarcocystis* species. Morphological characterization of these parasites includes principally the analysis of sarcocyst size and shape, morphometric parameters of bradyzoites located inside the sarcocyst, and the structure of the sarcocyst wall [1]. *Sarcocystis* species are distinguished by a combination of morphological analysis and sequence data of *18S* ribosomal DNA (rDNA) [1,4]. Significantly less frequently, the identification of these parasites is performed in definitive hosts by examining intestinal samples and feces of predators or scavengers [5]. *Sarcocystis* species cannot be distinguished by the morphology of sporocysts. Therefore, species of these parasites in the definitive host and environmental samples are identified by using nested PCR for the detection and sequencing of cytochrome c oxidase subunit I (*cox1*), *18S* rRNA, *28S* rRNA, and *ITS1* [6].

Until now, only two studies on *Sarcocystis* spp. diversity in water samples have been conducted due to the lack of validated methods [7,8]. In these studies, samples were collected from water sources located near villages on Tioman Island, Malaysia, where cases of muscular sarcocystosis were recorded. Sporocysts were isolated from water samples using the sucrose gradient method [9]. The nested PCR and Sanger sequencing of *18S* rRNA fragments allowed the identification of *S*. *nesbitti*, *S*. *singaporensis*, *Sarcosystis* sp. YLL-2013, and *Sarcocystis* sp. [7]. On the other hand, *S*. *nesbitti*, *S*. *singaporensis*, *S*. *zamani*, and several unnamed *Sarcocystis* species were defined by an improved *Sarcocystis* spp. identification method, relying on the next-generation sequencing (NGS) of *18S* rRNA and *28S* rRNA coding fragments [8]. Thus, water samples collected near pastures and human bathing areas could be used for epidemiology research aimed at detecting pathogenic species and those involving economically important animals as their hosts.

In the present study, we chose to examine the presence of eight *Sarcocystis* species (*S*. *bovifelis*, *S*. *cruzi*, *S*. *hirsuta*, *S*. *arieticanis*, *S*. *tenella*, *S*. *capracanis*, *S*. *bertrami*, and *S*. *miescheriana*) in water samples. Three species, *S. bovifelis*, *S. cruzi*, and *S. hirsuta*, form sarcocysts in muscles of cattle, *S. arieticanis* and *S. tenella* parasitize sheep, while *S*. *capracanis*, *S*. *bertrami*, and *S*. *miescheriana* are specific to goats, horses, and pig/wild boar, respectively. There is considerable confusion over classification of *Sarcocystis* species found in horses. Sarcocysts of *S*. *bertrami* and *S*. *fayeri* detected in muscles of horses show significant morphological differences [1]. However, *S*. *bertrami* and *S*. *fayeri* are the same species based on molecular markers, and *S*. *fayeri* should be considered a junior synonym of *S*. *bertrami* [10]. In the present study, we used *S*. *bertrami* to describe any *Sarcocystis* species from horses. So far *Sarcocystis* species composition in Lithuania was investigated only in cattle. It was shown that *S*. *bovifelis*, *S*. *cruzi* and *S*. *hirsuta* are the most common species in diaphragm muscles of cattle [11]. According to current knowledge, the other species selected in this work are most detected in sheep, goats, horses, and pigs in Europe [1].

The objective of the present study was to evaluate various sample preparation and conventional PCR methods that may aid in species-specific molecular identification of *Sarcocystis* residing in watersheds.

## 2. Materials and Methods

### 2.1. Sample Collection

**Figure 1:**
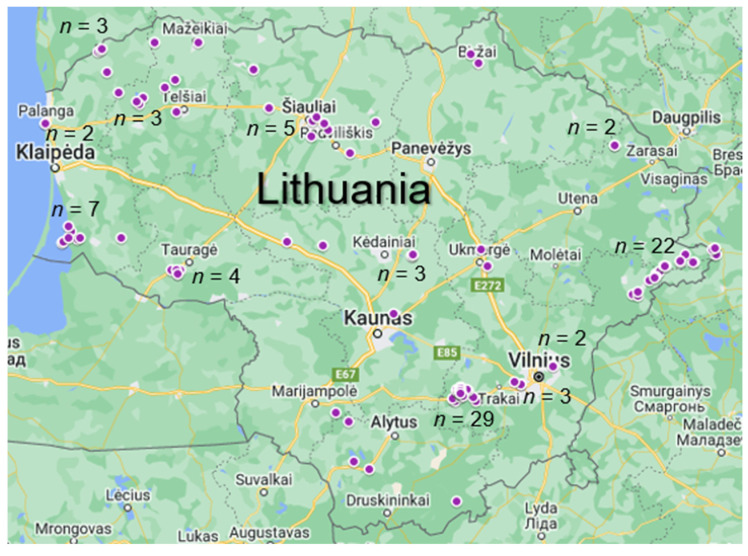
Water sampling sites in Lithuania in 2020.

Water samples (*n* = 114) were collected from different regions of Lithuania in 2020 and were analyzed for the presence of tested *Sarcocystis* spp. (Figure 1). Samples were collected during the summer, when the highest animal activity and migration rate is observed in Lithuania. Samples were gathered not only near pastures, but also near human bathing and remote wooded areas from various water sources such as ponds, canals, rivers, lakes, and lagoons. Water samples were collected up to one meter from the shore, gently stirring if taken from a standing water body. More than 20 L of water is usually collected for studies on parasitic protozoa in water [12,13]. Due to the relatively high *Sarcocystis* infection prevalence and intensity in farm animals from Lithuania [14], it was hypothesized that a smaller sample volume will be sufficient for the identification of these parasites in water samples. In this way, the conditions for transposition and laboratory examinations were facilitated.

To evaluate sample volume for testing, 3 different volumes of water samples (200 mL, 1 L, and 3 L) were collected in sterile containers and stored in portable coolers with ice batteries. Since water samples were collected in small-capacity vessels, sample collection and transportation were a simple and effortless process; we were able to collect up to 10–20 water samples per day. In the pilot study, 20 water samples of each volume (200 mL, 1 L, and 3 L) were collected and tested by nested PCR. For the first round of nested PCR, 20 samples were analyzed using three primer pairs SF1/SR8D, SF1/SR9, and SF1/SR12H, which were known to be suitable for amplification of the *cox1* gene of six of the tested *Sarcocystis* species (Table 1) [11,15,16,17,18,19]. Meanwhile, species-specific primers were applied to the second round of PCR. Only two samples were positive when a volume of 200 mL was used, while five samples were positive in both larger volume cases. It was decided that a 1 L volume of sample is appropriate for further large-scale analysis. The decision was made taking into consideration technical aspects of material processing at our laboratory. When 3 L of water was treated, it was more difficult to remove impurities such as algae, sand, and plant parts.

### 2.2. Isolation of Sporocysts and Genomic DNA Extraction

To isolate *Sarcocystis* spp. sporocysts, three different techniques, including sucrose gradient (specific gravity = 1.15) [9], sedimentation, and filtration, were used. During the pilot study, 35 samples were analyzed by each of these methods to select the one for the further examination of all collected samples. All three methods were performed at room temperature. For the sucrose density gradient concentration, the sample was centrifuged in 250 mL bottles at 800× *g* for 10 min, then the supernatant was discarded, and the precipitate was suspended in 50 mL of 2 M sucrose solution. The prepared sample was centrifuged for 10 min at 800× *g*, and after centrifugation, the upper part of the supernatant (5–10 mL) was transferred to a new 50 mL tube. The sample was washed with 45 mL of sterile water and centrifuged for 10 min at 800× *g*. The supernatant was discarded, and the pellet was suspended in a mixture of 3 mL of HBSS antibiotics. The sample was stored at 4 °C. For sedimentation concentration, the sample was centrifuged in 250 mL bottles at 5000× *g* for 25 min, then the supernatant was discarded, and the pellet was suspended in sterile water and transferred to 1.5 mL tubes. The prepared sample was centrifuged for 10 min at 14,000× *g*. After centrifugation, the supernatant was discarded, and the pellet was stored at −20 °C. To concentrate sporocysts by filtration, water samples were filtrated using a 1 mm pore metal sieve, then Whatman™ Qualitative Filter Paper Grade 4, and finally MF-Millipore^®^ 5 μm pore membranes. Membranes were washed in a sterile dish with 2 mL of distilled water. Concentrated samples were placed in sterile 2 mL tubes and stored at +4 °C.

The isolation of genomic DNA (gDNA) of *Sarcocystis* parasites from dry pellets (after sedimentation) was performed using the Genomic DNA Purification Kit (Thermo Fisher Scientific Baltics, Vilnius, Lithuania) and following the manufacturer’s protocol. The extraction of gDNA from water samples (after sucrose gradient and filtration) was accomplished using the GeneJET Genomic DNA Purification Kit (Thermo Fisher Scientific Baltics, Vilnius, Lithuania), according to the manufacturer’s recommendations. The maximum sample volume of 200 μL was selected according to the administration protocol. The resulting DNA samples were kept frozen at −20 °C until PCR was performed.

At the beginning of the study, the sucrose gradient method [9] and sediment collection from water were used for isolating sporocysts. PCR procedures were the same as described previously. After 35 water samples had been examined, it was observed that PCR fragments were more frequently obtained in samples using centrifugation at sucrose gradient (8/35, 22.9%) than sedimentation (3/35, 8.6%). Based on the results obtained, it was concluded that none of these methods is optimal for the isolation of sporocysts. It has been hypothesized that effective isolation of sporocysts may be hindered by a variety of impurities in water samples. As a result, it was decided to test the filtration of water samples. After analyzing 35 water samples by this method, the highest *Sarcocystis* spp. detection rate (14/35, 40.0%) was achieved. Consequently, this method was further applied to isolate sporocysts.

### 2.3. Identification of Sarcocystis Species Using PCR Procedures

Water samples were tested for the presence of eight *Sarcocystis* species (*S*. *bovifelis*, *S*. *cruzi*, *S*. *hirsuta*, *S*. *arieticanis*, *S*. *tenella*, *S*. *capracanis*, *S*. *bertrami*, and *S*. *miescheriana*) known to use agricultural species (cattle, sheep, goats, horses, and pigs) as intermediate hosts.

During the optimization of DNA fragment amplification, different conditions, and different types of conventional PCR, such as direct PCR, multiplex-nested PCR, semi-nested PCR, and nested PCR, were tested. Primers targeting the *cox1* gene were designed using Primer3Plus [20]. Some primers were chosen to be specific to several *Sarcocystis* spp., while others were specific to one *Sarcocystis* species (Table 1). The specificity of the primers was tested with negative, no template, and positive controls, i.e., DNA extracted from sarcocysts of the selected *Sarcocystis* species. Positive DNA controls of *Sarcocystis* species analyzed were accumulated during previous studies, confirmed by sequencing, and stored at the Laboratory of Molecular Ecology, Vilnius, Lithuania. Both positive and negative DNA controls were used in all PCR reactions.

Each PCR was performed in a final volume of 25 μL containing 0.5 μM of each primer (Invitrogen by Thermo Fisher Scientific, Waltham, MA, USA), 2.5 μL of dNTP mix (0.2 mM of each), 2.5 μL of 10× DreamTaq buffer with 20 mM MgCl_2_, 1.25 U of DreamTaq polymerase (Thermo Fisher Scientific Baltics, Vilnius, Lithuania), and 5 μL of genomic DNA and nuclease-free water. For nested PCR, 5 μL of genomic DNA was used for the first round, and 5 μL of products of the first round were then subjected to the second round of amplification. Conditions for nested PCR were as follows: initial denaturation at 95 °C for 5 min, followed by 30 (1st PCR) or 40 (2nd PCR) cycles of 94 °C for 45 s, 56−65 °C (depending on primers—see Table 1 for annealing temps for each primer set) for 45 s and 72 °C for 30−60 s (depending on the length of fragments (see Table 1 for extension time for each primer set)), and a final extension with one cycle at 72 °C for 10 min. PCR products were visualized by 1% agarose gel electrophoresis.

The selected PCR products were purified using the GeneJET PCR Purification Kit (Thermo Fisher Scientific Baltics, Vilnius, Lithuania) according to the manufacturer’s recommendations. Purified PCR fragments were sequenced in both directions using forward and reverse primers. The sequencing was performed using the Big-Dye^®^ Terminator v3.1 Cycle Sequencing Kit (Thermo Fisher Scientific, Vilnius, Lithuania) and the 3500 Genetic Analyzer (Applied Biosystems, Foster City, CA, USA). The obtained sequences were edited manually for ambiguously placed nucleotides and compared by BLAST analysis (http://blast.ncbi.nlm.nih.gov/, accessed on 22 March 2022).

### 2.4. Statistical Analysis

Statistical tests were performed using Quantitative Parasitology 3.0 software [21]. Sterne’s exact method was applied to compute the 95% confidence interval (CI) for the prevalence of identified *Sarcocystis* spp. [22]. Differences in the occurrence rates of the detected *Sarcocystis* species were evaluated using a Chi-squared test.

## 3. Results

### 3.1. Selection of PCR Conditions

We aimed to investigate conventional PCR methods for the identification of *Sarcocystis* parasites. As a result, several PCR approaches were analyzed in the present study, and the obtained results are summarized in Table 2. Briefly, to achieve the research objectives, direct, multiplex-nested, semi-nested, and nested PCRs were used. A series of primers (Table 1), theoretically amplifying single, several, or all eight species, were tested.

Finally, after sequencing obtained PCR fragments, we concluded that the application of nested PCR, using species-specific primers for both PCR rounds, is the best option. For each examined *Sarcocystis* spp., nested PCR with four different species-specific primers was used (Table 1). Having examined 35 samples, at least one species of *Sarcocystis* was detected in 34 samples (34/35, 97.1%). In addition, DNA fragments corresponding to the theoretical size were obtained using all species-specific primers. Therefore, this PCR type was used to analyze a total set of 114 samples.

### 3.2. Identification of Sarcocystis *spp.* from in Water Bodies

Overall, we obtained 274 PCR fragments. Of these, 73 theoretically representing 8 *Sarcocystis* spp. were sequenced. In total, we obtained four to twenty sequences for each species. The BLAST analysis showed that all of them represented tested species. To have an equal number of sequences, four sequences of each species were deposited in NCBI GenBank with Accession Numbers ON211315–ON211346 (Table 3). The obtained sequences were truncated by discarding the nucleotide sites, where DNA binds to the primers. Each set of four sequences generated by the same primer pair showed intraspecific genetic variability. Comparing the obtained sequences and those of the same *Sarcocystis* species available in GenBank, relatively large sequence similarity intervals were observed for *S*. *arieticanis* (92.3–99.4%) and *S*. *miescheriana* (93.9–99.5%). Low similarity values were established comparing the isolates identified in the current work with African isolates of *S*. *arieticanis* (92.3–93.5%; MH413047-48) and Asian isolates of *S*. *miescheriana* (93.9–95.3%; LC349977-80, MK867462-64). In general, interspecific, and intraspecific genetic variability values of tested *Sarcocystis* spp. did not overlap. Thus, the species-specific primers for nested PCR presented in Table 1 are suitable for the identification of eight *Sarcocystis* spp. (*S*. *bovifelis*, *S*. *cruzi*, *S*. *hirsuta*, *S*. *arieticanis*, *S*. *tenella*, *S*. *capracanis*, *S*. *bertrami*, and *S*. *miescheriana*).

### 3.3. Summary of Molecular-Based Sarcocystis Identification from Water Samples

**Figure 2:**
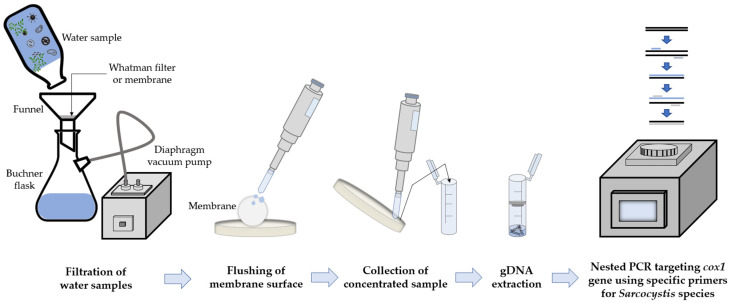
Scheme of molecular identification of *Sarcocystis* parasites from water samples.

During this work, suitable conditions for the identification of *Sarcocystis* spp. using farm animals as their intermediate hosts from water samples were selected (Figure 2). We used 1L of water sample for the detection of DNA of *Sarcocystis* spp. Impurities in water samples were removed by filtration using a 1 mm pore metal sieve, followed by Whatman™ Qualitative Filter Paper Grade 4. Sporocysts were collected on MF-Millipore^®^ 5 μm pore membranes and washed with 2 mL of sterile distilled water on a glass plate. Concentrated samples were transferred to sterile 2 mL Eppendorf tubes. The prepared and concentrated water sample was used for gDNA extraction employing the GeneJET Genomic DNA Purification Kit. Different types of PCR were tested during optimization: direct, multiplex-nested, semi-nested, and nested. Based on the results obtained, it was concluded that the most appropriate method for the identification of analyzed *Sarcocystis* spp. in water bodies is nested PCR, using species-specific primers in both rounds of PCR. The comparison of DNA sequences obtained confirmed that the developed primer sets presented in Table 1 were appropriate for the identification of eight species of *Sarcocystis* from farm animals.

### 3.4. Sarcocystis *spp.* Occurrence Rates in the Analyzed Water Samples

**Figure 3:**
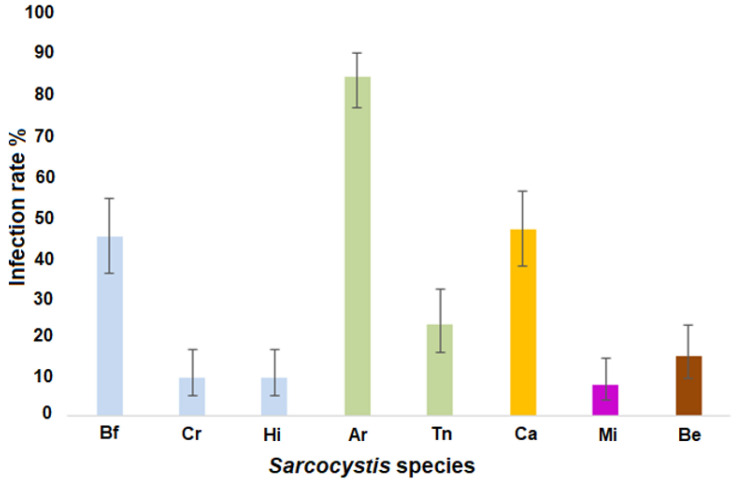
Prevalence of examined *Sarcocystis* species in Lithuanian water bodies in 2020 based on species-specific nested PCR. Bf—*S*. *bovifelis*, Cr—*S*. *cruzi*, Hi—*S*. *hirsuta*, Ar—*S*. *arieticanis*, Tn—*S*. *tenella*, Ca—*S*. *capracanis*, Mi—*S*. *miescheriana*, Be—*S*. *bertrami*.

Based on the applied molecular diagnostic technique, *Sarcocystis* spp. were detected in 111/114 analyzed water samples (97.4%; CI = 92.5–99.5). It should be noted that protozoans examined were confirmed in different water sources, ponds, canals, rivers, lakes, and lagoons. Comparing the analyzed species, the highest occurrence rate (96/114, 84.2%; CI = 76.4–90.0) was observed for *S*. *arieticanis* (Figure 3). The prevalence of *S*. *arieticanis* was significantly higher than that of *S*. *capracanis* (χ^2^ = 35.81, *p* < 0.00001), which was the second most frequently detected species (53/114, 46.5%; CI = 37.2–55.7). The third most often detected species was *S*. *bovifelis* (51/114, 44.7%; CI = 35.6–54.0). The prevalence of the fourth most detected species, *S*. *tenella* (26/114, 22.8%; CI = 15.7–31.5), was significantly lower than that of *S*. *capracanis* (χ^2^ = 14.12, *p* < 0.001) and that of *S*. *bovifelis* (χ^2^ = 12.26, *p* < 0.001). The occurrence rate of the remaining species was lower than 20%, i.e., 14.9% (17/114; CI = 9.3–22.6) for *S*. *bertrami*, 9.6% (11/114; CI = 5.1–16.6) for both *S*. *cruzi* and *S*. *hirsuta*, and 7.9% (9/114; CI = 4.0–14.4) for *S*. *miescheriana*. The prevalence of *S*. *tenella* was higher than that of *S*. *miescheriana* (χ^2^ = 9.75, *p* < 0.01), as well as *S*. *cruzi* and *S*. *hirsuta* (χ^2^ = 12.26, *p* < 0.01). The overall prevalence of *Sarcocystis* spp. using sheep as an intermediate host (88.6%) was significantly higher (χ^2^ = 32.73, *p* < 0.00001) than that of *Sarcocystis* spp. using cattle as an intermediate host (54.4%). Whereas no significant difference (*p* > 0.05) between the occurrence of *Sarcocystis* spp. employing cattle as an intermediate host and those employing goats as an intermediate host has been noticed. The number of *Sarcocystis* species per sample varied from one to five. Most commonly three *Sarcocystis* species (37 samples, 32.5%) were identified, while two species were detected in 34 samples (29.8%), one species was found in 23 samples (20.2%), four species were confirmed in 14 samples (12.3%) and five species were established in three samples (2.6%).

## 4. Discussion

In the present study, we aimed to select suitable conditions for rapid identification of *Sarcocystis* spp. in water samples. For this purpose, a sufficient amount of water was selected to evaluate the presence of these protozoan parasites in water samples. One liter was shown to be the suitable volume, in terms of sensitivity and technical factors of our laboratory. The chosen amount of a sample is considerably smaller as compared to that in other studies on the identification of protozoan parasites in water bodies. For instance, 100 L of water or more is taken for the detection of *Giardia* spp. and *Cryptosporidium* spp. [12,13,23]. The choice of high volumes of water is based on a low concentration of protozoan oocysts in water [24]. However, large quantities of water samples not only complicate the work in laboratories, but also require special containers and transport. Low concentration of protozoan parasites, morphological similarities of (oo)cysts, and various impurities (e.g., plant parts, sand, sludge, and different microorganisms) complicate the identification of these parasites in water samples. Thus, the identification of protozoan parasites from water often consists of several steps. Typically, the samples are first concentrated by filtration or centrifugation [24,25]. Due to the different sizes of parasitic protozoan oocysts (for example, cysts of *Cryptosporidium* spp. are about 4 × 6 μm, while *Sarcocystis* spp. are about 10 × 15 μm), it is impossible to concentrate parasites belonging to different genera during filtration [1,26]. Filtration is usually followed by purification using immunomagnetic separation and immunofluorescence microscopy [26]. During immunomagnetic separation, (oo)cysts are isolated using antibody-coated particles, and impurities that impede isolation and further identification are removed. However, the use of immunological and fluorescent methods often makes it impossible to accurately identify many species belonging to protozoan parasites because of their low concentration in water and morphological similarities of (oo)cysts. In the present study, we suggest using filters with different porosities to remove impurities, collect sporocysts on MF-Millipore^®^ 5 μm pore membranes, and wash with 2 mL of sterile distilled water.

Even though the *Sarcocystis* is one of the most abundant genera in the phylum Apicomplexa, consisting of more than 200 species, a reliable species identification system based on several nuclear and organelle markers has not been developed for these parasites yet [1]. It should be noted that the genus *Sarcocystis* is a heterogeneous group of organisms having complex evolutionary history depending on their host [27]. The largest DNA sequences database of *Sarcocystis* species has been accumulated for the *18S* rDNA [28]. However, this gene appeared to have low resolution power in separating closely related *Sarcocystis* species whose intermediate hosts are ungulates, including cervids, cattle, water buffalo (*Bubalus bubalis*), and sheep [29,30,31]. Some *Sarcocystis* species using cattle, sheep, goat, horse, and pig/wild boar as their intermediate host were characterized within nuclear *18S* rDNA, *28S* rDNA, *ITS1*, and *cox1* [15,30,32,33]. *Cox1* was found to be most suitable for the identification of *Sarcocystis* species using farm animals as their intermediate hosts [29,32,33]. In the present study, the compared 248–554 bp long *cox1* fragments appeared to be variable enough for the identification of the eight *Sarcocystis* species examined. Relatively high intraspecific variability values were observed when comparing the obtained sequences with those of African isolates of *S*. *arieticanis* [34] and Asian isolates of *S*. *miescheriana* [32]. In the current study, tested *Sarcocystis* species were mainly molecularly investigated in Europe and Asia [15,30,32,33]. Therefore, molecular characterization of *Sarcocystis* spp. from farm animals collected in different geographic regions and belonging to different animal breeds is necessary for accurate diagnosis of these species. In our research, the best results were obtained with the help of nested PCR and species-specific primers in both rounds of PCR. By contrast, in some cases, the use of primers specific for several *Sarcocystis* spp. resulted in the amplification of non-target *Sarcocystis* species (Table 2). Low specificity of primers can be explained by small interspecific differences in primer binding sites. In summary, the PCR results of the current study can be used to further develop *Sarcocystis* species identification techniques from environmental samples.

The present study is the first to identify *S*. *bovifelis*, *S*. *cruzi*, *S*. *hirsuta*, *S*. *arieticanis*, *S*. *tenella*, *S*. *capracanis*, *S*. *bertrami*, and *S*. *miescheriana* in environmental water samples. Furthermore, this is the first investigation devoted to diagnosing eight *Sarcocystis* species in water bodies. In the current work, using nested PCR and species-specific primers in both rounds, 274 DNA fragments were obtained; 73 of them were sequenced and showed the highest similarity with tested species. Since not all positive PCR fragments were sequenced, the obtained results should be considered with caution. Previously, up to three different *Sarcocystis* species were detected in water samples [7,8]. Using NGS, *S*. *nesbitti*, *S*. *singaporensis*, and *S*. *zamani* were identified, and based on the frequency of the presence, abundance, and identity of sequences, at least nine more individual but not yet formally described species were detected in the water samples analyzed in Malaysia [8]. The latter study showed that the diversity of *Sarcocystis* species has not been fully revealed yet.

Of the Lithuanian wild fauna, the European bison (Bison bonasus) can possibly act as an intermediate host of *S. bovifelis*, *S. cruzi*, and *S. hirsuta* [35,36], while *S. arieticanis*, *S. capracanis*, and *S. tenella* can form sarcocysts in muscles of European mouflon (Ovis aries musimon) [18]. However, populations of both wild animal species are limited to mainly central Lithuania and are composed of less than 500 individuals [37]. Thus, we assume that in Lithuania, the main intermediate hosts of the identified *Sarcocystis* species in the present work are farm animals.

According to the Department of Statistics of Lithuania, about 635,000 cattle and dairy cows, 152,000 sheep, 15,000goats, 13,000 horses, and 551,000 pigs were raised on Lithuanian farms in 2020 (https://osp.stat.gov.lt/lietuvos-aplinka-zeme-ukis-ir-energetika-2020/zemes-ukis/gyvulininkyste, accessed on 25 April 2022). The prevalence of *Sarcocystis* spp. and infection intensity in cattle, sheep, pigs, and horses were exhaustively examined in Lithuania [14]. The analysis of the esophagus, diaphragm, heart, neck, and jaw muscles showed high infection rates in sheep (100%) and cattle (88.0%), and moderate infection rates in horses (47.2%) and pigs (40.2%). The highest infection intensity was detected in sheep (Median, Md = 34.0), lower infection intensity was found in cattle (Md = 11.5) and pigs (Md = 11.50), and the lowest infection intensity in horses (Md = 2.0). Intense infection (>40 cysts per gram of muscle) was observed relatively often in sheep (44.9%), less frequently in cattle (19.1%), and occasionally in pigs (3.7%). The prevalence of different *Sarcocystis* species was performed only in the diaphragm muscles of cattle [11]. The analyzed diaphragm samples were most often infected with *S*. *cruzi* (96.1%), less frequently with *S*. *bovifelis* (71.6%) and *S*. *hirsuta* (30.4%), and the lowest with *S. hominis* (13.7%). The results obtained in this work are partially consistent with the abundance of tested farm animals and with the data of *Sarcocystis* spp. infection in muscles [11,14]. In the current study, we found a significantly higher infection prevalence of *Sarcocystis* spp. employing sheep as an intermediate host (88.6%) compared to cattle (54.4%). In addition, low infection rates were observed for *S*. *bertrami* (14.9%) and for *S*. *miescheriana* (7.9%), which are specific to horses and pigs, respectively. An attempt was made to identify *Sarcocystis hominis* in water samples by different conventional PCR methods using species-specific primers, but we were unable to detect them. The concentration of *S. hominis* in water bodies is considered to be too low to be identified by nested PCR. In previous morphological studies that we conducted [11], *S. hominis* was not detected by morphological methods in cattle carcasses. As a result, it was assumed that the concentration of *S. hominis* is very low, even in the cattle carcasses, so that few sporocysts enter the environment. However, the detection of *S*. *cruzi* in only 11 samples (9.6%) contradicts the data from the previous study. We assume that the primers developed for the identification of *S*. *cruzi* were not specific enough to some isolates of this species. In general, the results on infection rates of individual *Sarcocystis* species should be considered with caution. Hence, further development of molecular-based diagnosis of *Sarcocystis* spp. in water samples is required. In the future, to develop the identification of *Sarcocystis* spp. DNA from water samples, an attempt should be made to perform qPCR, which is a more sensitive and specific method. Furthermore, amplification of internal controls would improve the methodology for detecting *Sarcocystis* species in water samples.

## 5. Conclusions

This study presents the first identification of *Sarcocystis* species using farm animals as their intermediate hosts in water bodies. *Sarcocystis* spp. were detected in different water sources, such as ponds, canals, rivers, lakes, and lagoons. During the study, the volume of samples, the techniques for isolating sporocysts from water, and molecular detection procedures were investigated. Nested PCR using species-specific primers in both rounds of PCR gave the best results. The prevalence of *Sarcocystis* species analyzed significantly varied in the examined water samples. Based on the molecular method used, eight *Sarcocystis* species were identified, *S. cruzi* (9.6%), *S. bovifelis* (44.7%), *S. hirsuta* (9.6%), *S. tenella* (22.8%), *S. arieticanis* (84.2%), *S. capracanis* (46.5%), *S. miescheriana* (7.9%), and *S. bertrami* (14.9%). Thus, a convenient molecular method was developed for the identification of *Sarcocystis* parasites from farm animals in water samples. The suggested procedure can be used for epidemiological research and for tracking infection outbreaks.

## Figures and Tables

**Table 1:** Oligonucleotide primers used in this study for PCR procedures.

Species	Primers			Ta, °C	Extension Time, s	Product Size (bp)	Reference
	Name	Orientation	Sequence (5′–3′)				
Primers for Direct and Nested PCR							
*Sarcocystis* spp.	SF1	Forward	ATGGCGTACAACAATCATAAAGAA				[15]
	SR8D	Reverse	CATTGCCCATDACTACGCC	55	70	1072	[15]
	SR9	Reverse	ATATCCATACCRCCATTGCCCAT	60	70	1085	[16]
	SR12H	Reverse	AAATACCTTGGTGCCCGTAG	56	60	952	[17]
*S. bovifelis*	GaBfEF	Forward	ATCAACTTCCTAGGTACAGCGGTATT	56	45	523	[11]
	GaBfER	Reverse	CCACATCATTGGTGCTTAGTCTAGTA				[11]
*S. cruzi*	GaCrEF	Forward	GCTATGTATCTACTTACGGCAGGTATC	56	45	608	[11]
	GaCrER	Reverse	GAATATAATGGCCCAGGTAAATAATG				[11]
*S. hirsuta*	GaHiEF	Forward	GTTGTGCGGTATGAATTATCAACCT	56	45	513	[11]
	GaHiER	Reverse	GGTAAGAACTGGAATGGTTAATATCAG				[11]
*S. arieticanis*	GsSariF	Forward	TTCTTGGTATGGCTATTCTTGGACT	65	45	586	[18]
	GsSariR	Reverse	GATATGTCAATCCAGAGATCGGTAG				[18]
*S. tenella*	GsStenF	Forward	TACTCGGAGCGGTGAACTTCTTA	63	35	451	[18]
	GsStenR	Reverse	ATAGTCACGGCAGAGAAGTAGGAC				[18]
*S. capracanis*	GsScapF	Forward	AGCGGTAAACTTCCTGGGTACT	63	35	467	[18]
	GsScapR	Reverse	GCCTATCCAGTTGAATATCTTGGT				[18]
Primers for multiplex-nested PCR							
1st round							
*Sarcocystis* spp.	SF1	Forward	ATGGCGTACAACAATCATAAAGAA				[15]
*S. arieticanis*, *S*. *cruzi*	SsunR2	Reverse	GTGCCTCCCAGGCTGAAYAG	56	70	1055	[19]
*S. bertrami*, *S. tenella*, *S. capracanis*	SsunR1	Reverse	GTACCGCCCAGGCTGAAYAG	56	70	1055	[18]
*S. bovifelis*, *S. hirsuta*	SkatR	Reverse	CAGGCTGAACAGHABTACGA	56	70	1042	[19]
*S. miescheriana*	SmieF	Forward	ACGCTGTATGCACCACTGAG	56	45	658	Present study
	SmieR	Reverse	CTGAACAGCGCTACAAATGC				
2nd round							
*S*. *bertrami*	GsSberF	Forward	GTATGAACTGTCAACGGATGGAGTA	65	35	482	Present study
	GsSberR	Reverse	TCAACATTAGCGAGGTAAATACTATC				
*S. miescheriana*	GsSmieF	Forward	GTTCCTCGGTATTAGCAGCGTACT	65	35	509	Present study
	GsSmieR	Reverse	AGTTAAATATTTTAGTGCCCGTTGGA				
Primers for semi-nested PCR							
*S. bovifelis*	VoboF	Forward	GATCGGTATTACTGTTGCACTCATT	58	45	701	Present study
	VoboR	Reverse	AGGCCACATCATTGGTGCTTA				
	GaBfEF	Forward	ATCAACTTCCTAGGTACAGCGGTATT	57	35	521	[11]
*S. tenella*	VoteF1	Forward	AGCGGTGAACTTCTTAGGAACC	59	35	526	Present study
	VoteR	Reverse	AATAATCCGCTGTTAACGTATGC				Present study
	VoteF2	Forward	CATTGTAATGCTCCTCGACGATA	59	30	401	Present study
*S. capracanis*	VocaF	Forward	GTAAACTTCCTGGGTACTGTGCTGT	60	35	526	Present study
	VocaR1	Reverse	CCAGTAATCCGCTGTCAAGATAC				Present study
	VocaR2	Reverse	AGTACCCATCACGGTGCCTATC	63	35	500	Present study
Species-specific primers for nested PCR							
*S. bovifelis*	V2bo1	Forward	AACTTCCTAGGTACAGCGGTATTCG	60	40	556	Present study
	V2bo2	Reverse	TGAACAGCAGTACGAAGGCAAC				Present study
	V2bo3	Forward	ATATTTACCGGTGCCGTACTTATGTT	60	30	410	Present study
	V2bo4	Reverse	GCCACATCATTGGTGCTTAGTCT				Present study
*S. cruzi*	V2cr1	Forward	TACAATGTGCTGTTTACGCTCCA	57	50	776	Present study
	V2cr2	Reverse	GCAATCATGATAGTTACGGCAGA				Present study
	V2cr3	Forward	ACCATCCTGTTCTGTGGTGCTATG	65	30	298	Present study
	V2cr4	Reverse	AAACTACTTTACTGCCTACGGTACTC				Present study
*S. hirsuta*	V2hi5	Forward	TATGTTGGTTCTGCCGAAGTCAT	60	45	686	Present study
	V2hi6	Reverse	GGTATGGCAATCATTATGGTTACAG				Present study
	V2hi7	Forward	GCACCGTAATATTTCAGGGATGT	60	30	299	Present study
	V2hi8	Reverse	AACCTGCTTGCCGGAGTAAGTA				Present study
*S. arieticanis*	V2arie1	Forward	CTCTTTGCCGTAGATTCGCTAGTTA	63	55	884	Present study
	V2arie2	Reverse	CAAAGATCGGTAGATATCCAATGC				Present study
	V2arie3	Forward	TAGTTCTTGGCCTGGCTATTCTT	59	25	371	Present study
	V2arie4	Reverse	CTGACCTCCAAAAACTGGCTTAC				Present study
*S. tenella*	V2te1	Forward	GAGCGGTGAACTTCTTAGGAACC	60	40	537	Present study
	V2te2	Reverse	CCCAATAATCCGCTGTTAACGTA				Present study
	V2te3b	Forward	ATTGTAATGCTCCTCGACGATATG	57	30	314	Present study
	V2te4	Reverse	ATAGTCACGGCAGAGAAGTAGGAC				Present study
*S. capracanis*	VocaF	Forward	GTAAACTTCCTGGGTACTGTGCTGT	60	40	531	Present study
	VocaR1	Reverse	CCAGTAATCCGCTGTCAAGATAC				Present study
	V2cap3	Forward	ATACCGATCTTTACGGGAGCAGTA	63	30	330	Present study
	V2cap4	Reverse	GGTCACCGCAGAGAAGTACGAT				Present study
*S. bertrami*	V2ber1	Forward	GTATGAACTGTCAACGGATGGAGTA	58	60	883	Present study
	V2ber2	Reverse	AGAAGCCATGTTCGTGACTACC				Present study
	V2ber3	Forward	GTACTACCTCCTTCCAGTCGGTTC	57	40	600	Present study
	V2ber4	Reverse	CGGGTATCCACTTCAAGTCCAG				Present study
*S. miescheriana*	V2mie1	Forward	TGCTGCGGTATGAACTATCTACCT	61	60	922	Present study
	V2mie2	Reverse	GCCCAGAGATCCAAATCCAG				Present study
	V2mie3	Forward	CTTGGTTCAACGTTACTCCTCCA	61	30	474	Present study
	V2mie4	Reverse	CTTCGATCCAGCTGAACTAAAGC				Present study

SF1 is specific to genus *Sarcocystis*. SR8D, SR9, and SR12H are specific to some of the tested species or to some isolates of analyzed species. The remaining primer pairs were designed to be specific to certain *Sarcocystis* species. R = A or G, D = A or G or T2.4. PCR product purification, sequencing, and sequence analysis.

**Table 2:** Comparison of the used PCR approaches.

Method	Sporocysts Isolation	gDNA Extraction	Number of Water Samples (n)	Positive Samples *	Cases		Species Detected ****
					Positive **	False Positive ***	
Direct PCR (species-specific primers)	Sucrose gradient	“GeneJET Genomic DNA Purification Kit”	20	0 (0.0%)	0	0	0
Nested PCR (species-specific primers only in 2nd round of PCR)	Sucrose gradient	“GeneJET Genomic DNA Purification Kit”	35	8 (22.9%)	12	2	2
	Sedimentation	“Genomic DNA Purification Kit”	35	3 (8.6%)	4	1	2
	Filtration	“GeneJET Genomic DNA Purification Kit”	35	14 (40.0%)	15	1	3
Multiplex-nested PCR (species-specific primers only in 2nd round of PCR)	Filtration	“GeneJET Genomic DNA Purification Kit”	35	7 (20.0%)	10	3	2
Semi-nested PCR (species-specific primers)	Filtration	“GeneJET Genomic DNA Purification Kit”	35	0 (0.0%)	0	0	0
Nested PCR (species-specific primers in both rounds)	Filtration	“GeneJET Genomic DNA Purification Kit”	35	34 (97.1%)	84	0	8

* The number of samples in which at least one of targeted *Sarcocystis* species was identified. ** The number of positive PCR fragments. *** Based on sequencing the number of incorrectly identified species. **** Based on sequencing the number of targeted species identified in samples.

**Table 3:** Genetic identification of *Sarcocystis* spp. detected in watershed samples using nested PCR.

Species	GenBank Acc. No. (Length, bp)	Sequence Similarity, %		
		Comparing Obtained Sequences with Each Other	Comparing Obtained Sequences and Those of the Same Species Available in GenBank	Comparing Obtained Sequences with Those of Most Closely Related Species
*S. bovifelis*	ON211315-18 (361)	99.7–100	99.5–100	*S*. *bovini* 93.1–94.5
*S. cruzi*	ON211319-22 (248)	98.8–100	98.0–99.6	*S*. *alceslatrans* 85.1–86.8
*S. hirsuta*	ON211323-26 (254)	99.6–100	98.4–100	*S*. *buffalonis* 92.8–93.2
*S. arieticanis*	ON211327-30 (325)	99.7–100	92.3–99.4	*S*. *hircicanis* 86.2–87.4
*S. tenella*	ON211331-34 (263)	99.2–100	96.2–100	*S*. *capracanis* 91.3–93.2
*S. capracanis*	ON211335-38 (284)	99.7–100	96.8–99.7	*S*. *tenella* 90.4–92.9
*S. bertrami*	ON211339-42 (554)	99.1–99.8	97.5–99.8	*S*. *bovifelis* 77.0–78.4
*S. miescheriana*	ON211343-46 (428)	99.5–100	93.9–99.5	*S*. *rangiferi* 79.6–80.2

## Data Availability

Data supporting the conclusions of this article are included in the article. The sequences generated in the present study were submitted to the GenBank database under Accession Numbers ON211315–ON211346.
